# HOTAIRM1 knockdown reduces MPP^+^-induced oxidative stress injury of SH-SY5Y cells by activating the Nrf2/HO-1 pathway

**DOI:** 10.1515/tnsci-2022-0296

**Published:** 2023-07-25

**Authors:** Hui-Yu Dai, Ming-Xiu Chang, Ling Sun

**Affiliations:** Department of Neurology, The Fourth Affiliated Hospital of Harbin Medical University, Harbin, Heilongjiang Province, China; Department of Neurology, The Fourth Affiliated Hospital of Harbin Medical University, Harbin, Heilongjiang Province, China

**Keywords:** Parkinson’s disease, HOXA transcript antisense RNA myeloid-specific 1,1-methyl-4-phenylpyridonium, Nrf2/HO-1, oxidative stress

## Abstract

**Objective:**

Parkinson’s disease (PD) is the second most common neurodegenerative disease with complex pathogenesis. Although HOXA transcript antisense RNA myeloid-specific 1 (HOTAIRM1) is upregulated in PD, its exact role in HOTAIRM1 is seldom reported. The purpose of this study is to research the effect of HOTAIRM1 on 1-methyl-4-phenylpyridonium (MPP^+^)-induced cytotoxicity and oxidative stress in SH-SY5Y cells.

**Methods:**

SH-SY5Y cells were treated with MPP^+^ at various concentrations or time points to induce SH-SY5Y cytotoxicity, so as to determine the optimal MPP^+^ concentration and time point. HOTAIRM1 expression upon MPP^+^ treatment was analyzed through qRT-PCR. Next, HOTAIRM1 was downregulated to observe the variance of SH-SY5Y cell viability, apoptosis, oxidative stress-related indexes, and protein levels of the Nrf2/HO-1 pathway. In addition, rescue experiments were carried out to assess the role of Nrf2 silencing in HOTAIRM1 knockdown on MPP^+^-induced oxidative stress in SH-SY5Y cells.

**Results:**

MPP^+^ treatment-induced cytotoxicity and upregulated HOTAIRM1 expression in SH-SY5Y cells in a dose- and time-dependent manner. Mechanically, HOTAIRM1 knockdown enhanced cell viability, limited apoptosis, and oxidative stress, therefore protecting SH-SY5Y cells from MPP^+^-induced SH-SY5Y cytotoxicity. On the other hand, HOTAIRM1 knockdown activated the protein levels of Nrf2 and HO-1. Nrf2 silencing could counteract the neuroprotective effect of HOTAIRM1 knockdown on *in vitro* PD model.

**Conclusion:**

Our data demonstrated that HOTAIRM1 knockdown could inhibit apoptosis and oxidative stress and activated the Nrf2/HO-1 pathway, therefore exerting neuroprotective effect on the PD cell model.

## 1 Introduction

Parkinson’s disease (PD) is a kind of neurodegenerative disease, and it could eventually lead to degraded life quality to individuals and mounting burden on medical resources to society [1]. Traumatic brain damage, excessive smoking, pesticide exposure, medication use history, and lack of exercises are significant factors that aggravate PD syndrome [2]. Functionally, oxidative stress injury is prominently involved in PD pathogenesis and progression [3]. Apoptosis is prominently implicated in PD as it coordinates cellular homeostasis and dopaminergic neuron activity, and it is further induced upon 1-methyl-4-phenylpyridonium (MPP^+^) addition [4,5]. Moreover, it is indicated in several previous studies that MPP^+^-induced *in vitro* PD model could influence a variety of biological behaviors including inflammation, apoptotic reaction, and oxidative damage in PD progression [6,7,8], suggesting that establishing MPP^+^-induced *in vitro* PD model could be helpful in studying PD. From now on, PD can be relieved by appropriate usage of medication and physicotherapeutics, but a permanent and thorough treatment for PD remains vacant [9]. Against this background, possible biomarkers associating with neuroprotection and oxidative stress injury in PD are in urgent need [10].

Long non-coding RNAs (lncRNAs) participate in different mechanisms compassing apoptosis, nerve injury, and oxidative stress damage in PD progression [11]. Emerging evidence has reported that aberrant expression of lncRNA HOXA transcript antisense RNA myeloid-specific 1 (HOTAIRM1) is found in multiple human diseases, including cancers, sepsis, and progressive bone disorders [12,13,14]. HOTAIRM1 expression is higher in individuals with PD than that in healthy ones [15]. Remarkably, it was documented that HOTAIRM1 enhanced MPP^+^-elicited apoptosis and oxidative stress damage in neuroblastoma [16]. It is documented that the interaction between HOTAIRM1 and some transcription factors could influence different human disorders [17]. Nuclear factor E2-related factor 2 (Nrf2), as a famous transcription factor, modulates cellular growth, balance, and biological activities and gene metabolism, and it could cooperate with a variety of molecules to affect disease progression [18]. Interestingly, Nrf2 restricts oxidative stress and renders neuroprotection on tissues with PD, making it an attractive target for PD attenuation [19]. Elaborate knowledge about the network of Nrf2 and its downstream cytokines is essential to the research on PD development [20]. Heme oxygenase-1 (HO-1), a type of protein that is dependent on Nrf2, could regulate oxidative stress and apoptosis, and the neuroprotective property of the Nrf2/HO-1 pathway greatly retards cytotoxicity of many age-related human diseases [21]. At present, the relation between HOTAIRM1 and Nrf2 is still to be explored. Thus, *in vitro* PD model was established in SH-SY5Y cells with MPP^+^ treatment to determine the dynamic expression changes in HOTAIRM1. Afterwards, the effect of HOTAIRM1 knockdown on MPP^+^-induced cellular oxidative stress damage is investigated, and the crosstalk of HOTAIRM1 and the Nrf2/HO-1 pathway is further researched, therefore providing certain ideas for finding therapeutic targets for PD.

## 2 Materials and methods

### 2.1 Cell culture

Human neuroblastoma cells (SH-SY5Y, Shanghai Institute of Biochemistry and Cell Biology, Chinese Academy of Sciences, Shanghai, China) were cultured in Dulbecco’s modified Eagle medium (Gibco, Grand Island, NY, USA) consisting of 10% fetal bovine serum (Gibco) and 1% penicillin and streptomycin (Invitrogen, Thermo Fisher Scientific Inc., Waltham, MA, USA) in a sterile cell incubator with 5% CO_2_ at 37°C. When the cell confluence reached 80%, the cells were digested with trypsin and the well-growing passage three cells were collected for further experiments.

### 2.2 *In vitro* PD cell model establishment

MPP^+^ (No. D048, Sigma-Aldrich, Merck KGaA, Darmstadt, Germany) was dissolved in the cell culture medium, and when the final concentration was adjusted to 1 mmol/L, the medium was filtered and sterilized through a membrane (0.22 μm) and stored in a refrigerator at 4°C. SH-SY5Y cells in the exponential growth phase were inoculated into 96-well plates (1 × 10^4^ cells per well) for 12 h. MPP^+^ at different final concentrations (0, 0.25, 0.5, 1, and 2 mM) was allowed to react in the plates for 24 h, while 1 mM MPP^+^ reacted in the plates for different times (0, 12, 24, and 48 h). After the reagents were added according to the instructions of cell counting kit-8 (CCK-8) kit (Beyotime Biotechnology Co., Ltd, Shanghai, China), the optical density value at 450 nm wavelength of the cells treated with different concentrations of MPP^+^ was measured using a microplate reader for the calculation and analysis of cell viability, with 0 mM as the control.

### 2.3 Cell grouping and treatment

*In vitro* PD model was established using 1.0 mM MPP^+^-induced SH-SY5Y cells. First, cells in the exponential growth phase were allocated into the control group (SH-SY5Y cells with no MPP^+^ treatment), the MPP^+^ group (SH-SY5Y cells treated by 1.0 mM MPP^+^ for 24 h), the small interfere (si)-negative control (NC) (Shanghai GenePharma Co., Ltd, Shanghai, China) group (SH-SY5Y cells treated by 1.0 mM MPP^+^ for 24 h after the 24 h of si-NC transfection), and the si-HOTAIRM1 (GenePharma) group (SH-SY5Y cells treated by 1.0 mM MPP^+^ for 24 h following the 24 h of si-HOTAIRM1 transfection). To verify the role of Nrf2/HO-1 in *in vitro* PD model, SH-SY5Y cells were transfected by Nrf2-siRNA (GenePharma) for 24 h, which was followed by the 24 h of MPP^+^ treatment. When cell confluence reached 70–80%, transfection was conducted following the instructions of Lipofectamine 3000 (Invitrogen), and transfection efficiency was detected by quantitative real-time polymerase chain reaction (qRT-PCR).

### 2.4 CCK-8 assay

Cells were seeded into the 96-well plates (1 × 10^4^ cells per well) and incubated at a cell incubator for 24 h. Cell medium was discarded 4 h before the assay and cells were subject to three washes in phosphate-buffered saline (PBS). Subsequently, 10 μL of CCK-8 reagent was supplemented into each well for the 4-h incubation. Then, the optical density value at a wavelength of 450 nm was measured using a microplate reader to determine cell viability. The experiment was performed three times.

### 2.5 Terminal deoxynucleotidyl transferase-mediated dUTP nick end labeling (TUNEL) assay

SH-SY5Y apoptosis was assessed. After the culture, SH-SY5Y cells were rinsed using PBS and then fixed with 4% paraformaldehyde in PBS for 1 h. Next, cells were permeabilized by 0.1% Triton X-100 (Sigma) for 6 min and were marked and allowed to react with TUNEL reaction mixture devoid of light in 5% CO_2_ and 95% air at 37°C for 1 h. Afterwards, cells were cleaned with PBS and incubated with 4′,6-diamidino-2-phenylindole (DAPI; Sigma) for 10 min at room temperature. After another PBS wash, cell samples were photographed under a fluorescence microscope (Olympus, Tokyo, Japan). Apoptotic rate was calculated as the percentage of number of TUNEL-positive cells (green fluorescence)/total number of cells (DAPI-stained nuclei) × 100%. The experiment was performed three times.

### 2.6 Oxidative stress index assessment

The cells were collected, and the cell culture supernatant was harvested. Afterwards, reactive oxygen species (ROS) level, lactate dehydrogenase (LDH) activity, superoxide dismutase (SOD) activity, and malondialdehyde (MDA) level were measured based on the instructions of the ROS assay kit (S0033S), the LDH cytotoxicity assay kit (C0016), the SOD assay kit (S0101S), and the MDA assay kit (S0131S), respectively (all from Beyotime). The experiment was repeated three times.

### 2.7 qRT-PCR

The TRIzol reagent (Invitrogen) was appointed to extract total RNA in SH-SY5Y cells. To assess HOTAIRM1 expression, total RNA was adversely transcribed to cDNA with the cDNA kit (Thermo Fisher Scientific Inc., Waltham, MA, USA). HOTAIRM1 expression was determined using the PrimeScript RT-PCR kit (Roche, Basel, Switzerland), with glyceraldehyde-3-phosphate dehydrogenase as the internal reference. Primer sequence is shown in Table 1. Fluorescent qRT-PCR was performed by means of ABI7500 qPCR instrument (7500; ABI, Inc., Foster City, CA, USA), and the reaction conditions included 40 cycles of initial denaturation at 95°C for 10 min, denaturation at 95°C for 10 s, annealing at 60°C for 20 s, and extension at 72°C for 34 s. Relative quantification method (2^−ΔΔCT^ method) was appointed to measure relative transcription expression of target genes. The formula was as follows: ΔΔCT = ΔCt experimental group – ΔCt control group, ΔCt = Ct target gene – Ct internal reference. Ct is the number of amplification cycles required for the real-time fluorescence intensity of the reaction to reach the set threshold, in which amplification increases in a logarithmic phase. Each procedure was conducted for three times.

**Table 1:** Primer sequence of qRT-PCR

Gene	Sequence (5′–3′)
HOTAIRM1	F: CCCACCGTTCAATGAAAG
	R: GTTTCAAACACCCACATTTC
GAPDH	F: GTGGACCTGACCTGCCGTCT
	R: GGAGGAGTGGGTGTCGCTGT

Note: qRT-PCR, quantitative real-time polymerase chain reaction; HOTAIRM1, HOXA transcript antisense RNA myeloid-specific 1; GAPDH, glyceraldehyde-3-phosphate dehydrogenase; F, forward; R, reverse.

### 2.8 Western blot analysis

Cells from different groups were harvested and lysed with cell lysis solution containing protease inhibitor. The lysate was transferred to a 1.5 mL centrifuge tube and then centrifuged at 12,000*g* for 10 min at 4°C. The supernatant was extracted and the protein concentration was determined using the bicinchoninic acid protein quantitation kit (Boster Biological Technology Co., Ltd, Wuhan, Hubei, China). Protein was separated using sodium dodecyl sulfate–polyacrylamide gel electrophoresis and then transferred onto polyvinylidene fluoride membranes, which were then sealed with 5% skim milk for 2 h and incubated with diluted primary antibodies (1:1,000; all from Cell Signaling Technology, Danvers, MA, USA): B-cell lymphoma-2 (Bcl-2, #4223), Bcl-2-associated X (Bax, #2772), Nrf2 (#33649), HO-1(#70081), and β-actin (#4967) at 4°C overnight, followed by the incubation with horseradish peroxidase-labeled goat anti-rabbit secondary antibody (#7077, 1:2,000; Cell Signaling Technology) for 1 h at room temperature. Subsequently, membranes were incubated with enhanced chemiluminescence (ECL) assay reagent (EMD Millipore Corporation, Billerica, MA, USA) for 1 min. When the excess ECL reagents were removed, membranes were sealed and then developed and fixed with 5–10 min of X-ray film exposure in the dark. ImageJ software (National Institutes of Health, Bethesda, MD, USA) was applied to quantify the gray value of bands from each group, with β-actin as the internal reference, and the ratio of the gray value of the target band to that of the internal reference band was applied to calculate the expression of the target protein. Each procedure was repeated three times.

### 2.9 Statistical analysis

GraphPad Prism8.0 (GraphPad Software Inc., San Diego, CA, USA) was employed for data analysis and graphing. The results were displayed in mean ± standard deviation. The unpaired *t*-test was employed for comparison analysis between two independent groups, one-way or two-way analysis of variance was appointed for comparison analysis among multiple groups, and Tukey’s multiple comparisons test was used for the post-test of data. The *p* value was attained using a two-tailed test and *p* < 0.05 indicated a significant difference.

## 3 Results

### 3.1 HOTAIRM1 is overexpressed in MPP^+^-induced SH-SY5Y cells

**Figure 1:**
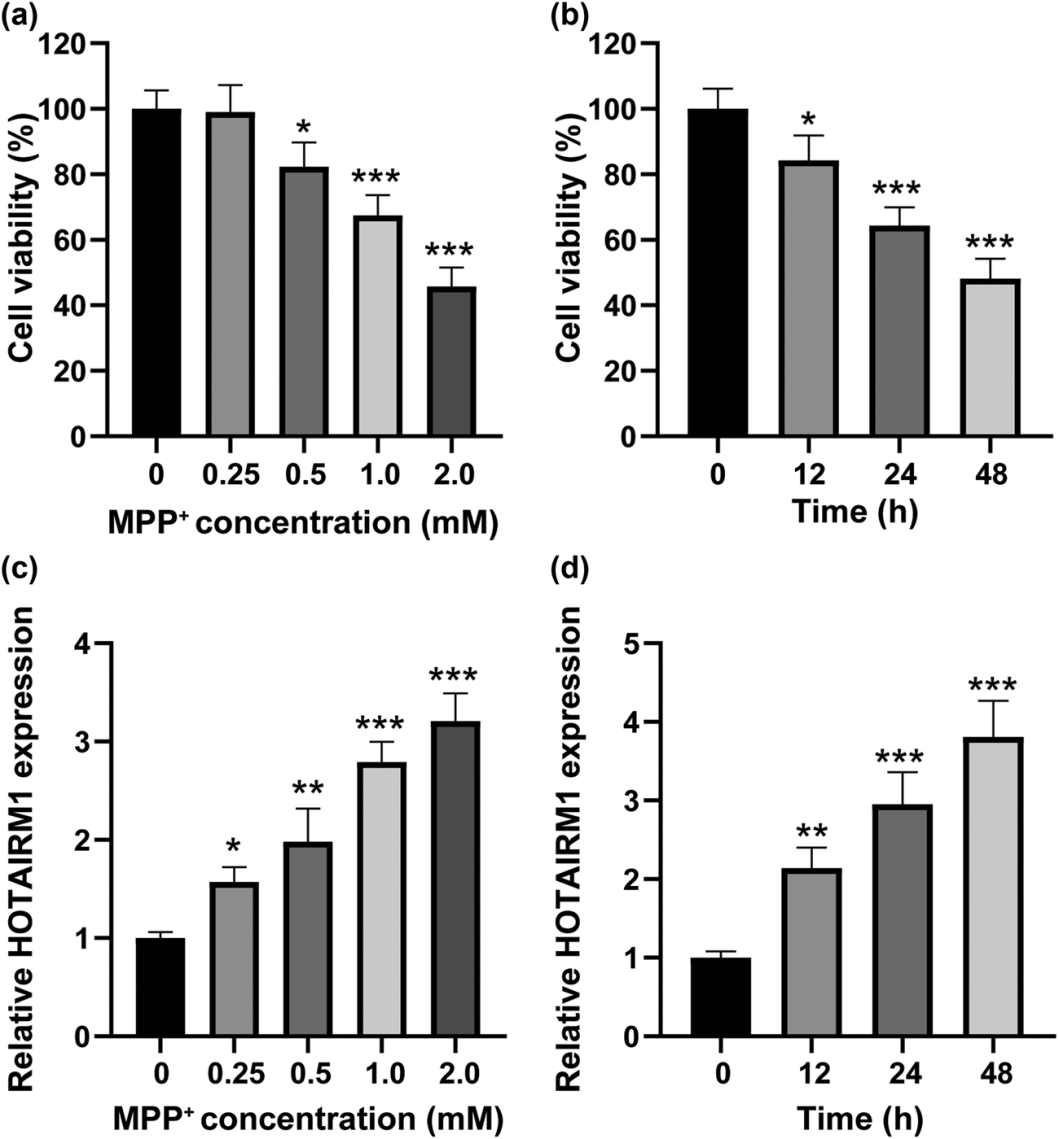
HOTAIRM1 is overexpressed in MPP^+^-induced SH-SY5Y cells. (a) SH-SY5Y cells were treated with MPP^+^ at various concentrations (0, 0.25, 0.5, 1.0, and 2.0 mM) for 24 h and dose-dependent cytotoxicity induced by MPP^+^ was assessed by CCK-8 assay. (b) SH-SY5Y cells were treated with 1 mM MPP^+^ for 0, 12, 24, and 48 h, respectively, and time-dependent cytotoxicity induced by MPP^+^ was evaluated by CCK-8 assay. (c) Dose-dependent HOTAIRM1 overexpression induced by MPP^+^ was measured by qRT-PCR. (d) Time-dependent HOTAIRM1 overexpression induced by MPP^+^ was determined by qRT-PCR. *p < 0.05, ***p < 0.001.

SH-SY5Y cells were treated with MPP^+^ at various concentrations (0, 0.25, 0.5, 1.0, and 2.0 mM) for 24 h before cytotoxicity induced by MPP^+^ was assessed. And the results indicated that SH-SY5Y cells were evidently destroyed upon 0.5, 1.0, and 2.0 mM MPP^+^ treatment (all *p* < 0.05, Figure 1(a)). Furthermore, in SH-SY5Y cells that received 1.0 mM MPP^+^ treatment, cellular destruction was exacerbated for 12 h or longer (all *p* < 0.05, Figure 1(b)). To validate HOTAIRM1 expression in the *in vitro* PD model, qRT-PCR was performed, after which it was discovered that HOTAIRM1 expression was upregulated in SH-SY5Y cells treated with 0.25 mM or higher concentrations of MPP^+^ (all *p* < 0.05, Figure 1(c)). Besides, HOTAIRM1 was overexpressed in SH-SY5Y cells that received 1.0 mM MPP^+^ treatment for 12 h or longer (all *p* < 0.05, Figure 1(d)). In summary, MPP^+^-induced SH-SY5Y cytotoxicity and HOTAIRM1 upregulation in SH-SY5Y cells exhibited a dose- and time-dependent manner.

### 3.2 HOTAIRM1 knockdown strengthens MPP^+^-induced SH-SY5Y cell viability

**Figure 2:**
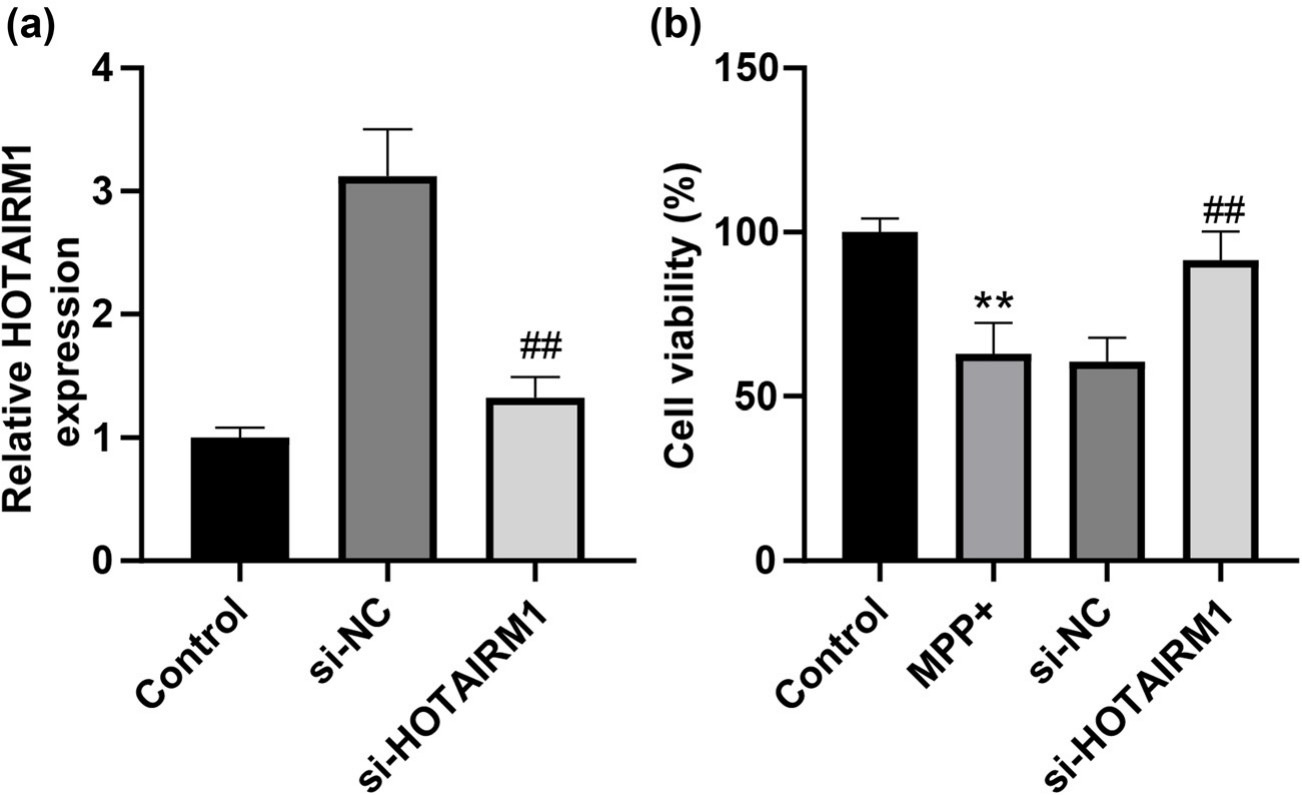
HOTAIRM1 knockdown strengthens MPP^+^-induced SH-SY5Y cell viability. si-NC and si-HOTAIRM1 were transfected into SH-SY5Y cells for 24 h, after which cells were stimulated by 1.0 mM MPP^+^ for 24 h. (a) HOTAIRM1 expression in SH-SY5Y cells was evaluated via qRT-PCR. (b) SH-SY5Y cell viability was determined by CCK-8 assay. Compared with the control group, **p < 0.01; compared with the si-NC group, ^##^ *p* < 0.01.

To figure out the effect of HOTAIRM1 on MPP^+^-induced SH-SY5Y cell viability, si-NC or si-HOTAIRM1 was transfected into SH-SY5Y cells for 24 h, after which cells were stimulated by 1.0 mM MPP^+^ for 24 h and then analyzed by qRT-PCR, which revealed that compared with the si-NC group, the si-HOTAIRM1 group presented decreased HOTAIRM1 expression in SH-SY5Y cells (*p* < 0.001, Figure 2(a)). Our data suggested that MPP^+^ treatment sabotaged SH-SY5Y cell viability, which was reversed by HOTAIRM1 knockdown (*p* < 0.01, Figure 2(b)), indicating that HOTAIRM1 knockdown could protect SH-SY5Y cells from MPP^+^-induced SH-SY5Y cytotoxicity.

### 3.3 HOTAIRM1 knockdown inhibits MPP^+^-induced SH-SY5Y cell apoptosis

**Figure 3:**
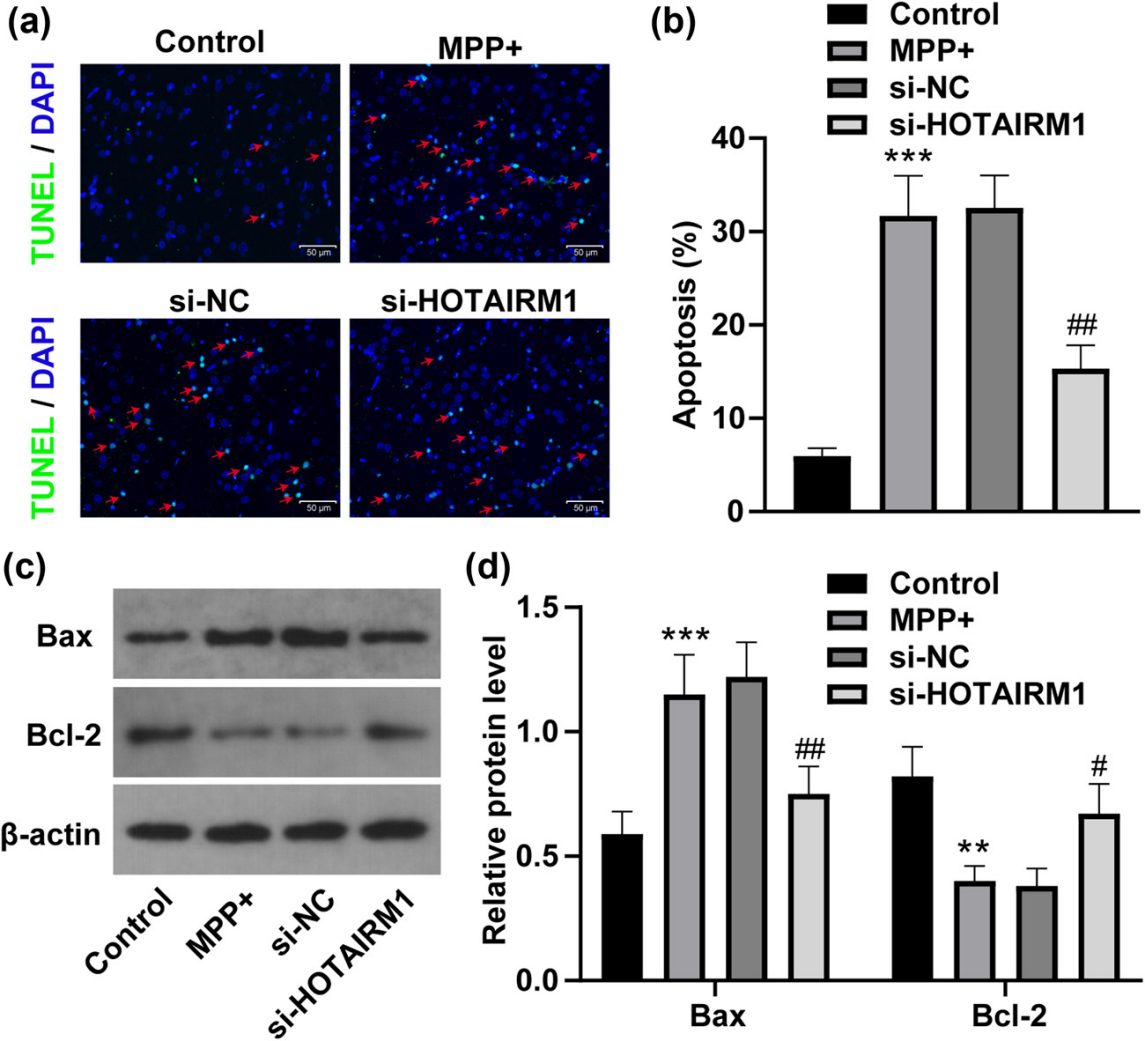
HOTAIRM1 knockdown inhibits MPP^+^-induced SH-SY5Y cell apoptosis. SH-SY5Y cells were transfected with si-NC and si-HOTAIRM1MPP^+^ for 24 h before cells were challenged by 1.0 mM MPP^+^ for 24 h. (a) TUNEL assay was conducted to assess apoptosis (red arrow indicating apoptosis). (b) Apoptotic analysis of (a). (c) The gray values of Bax and Bcl-2 were analyzed via western blot analysis. (d) Statistic analysis of the gray values of Bax and Bcl-2 in (c). Compared with the control group, **p < 0.01, ***p < 0.001; compared with the si-NC group, ^#^ *p* < 0.05, ^##^ *p* < 0.01.

Next, the role of HOTAIRM1 in MPP^+^-induced SH-SY5Y cell apoptosis was researched. TUNEL assay found that MPP^+^ treatment accelerated SH-SY5Y cell apoptosis while si-HOTAIRM1 decelerated it (all *p* < 0.01, Figure 3(a) and (b)). Western blot analysis exhibited that the upregulated Bax level and downregulated Bcl-2 level in SH-SY5Y cells treated by MPP^+^ were reversed by HOTAIRM1 knockdown (all *p* < 0.05, Figure 3(c) and (d)). Therefore, HOTAIRM1 knockdown inhibited MPP^+^-induced SH-SY5Y cell apoptosis.

### 3.4 HOTAIRM1 knockdown alleviates MPP^+^ induced SH-SY5Y cell oxidative stress injury

**Figure 4:**
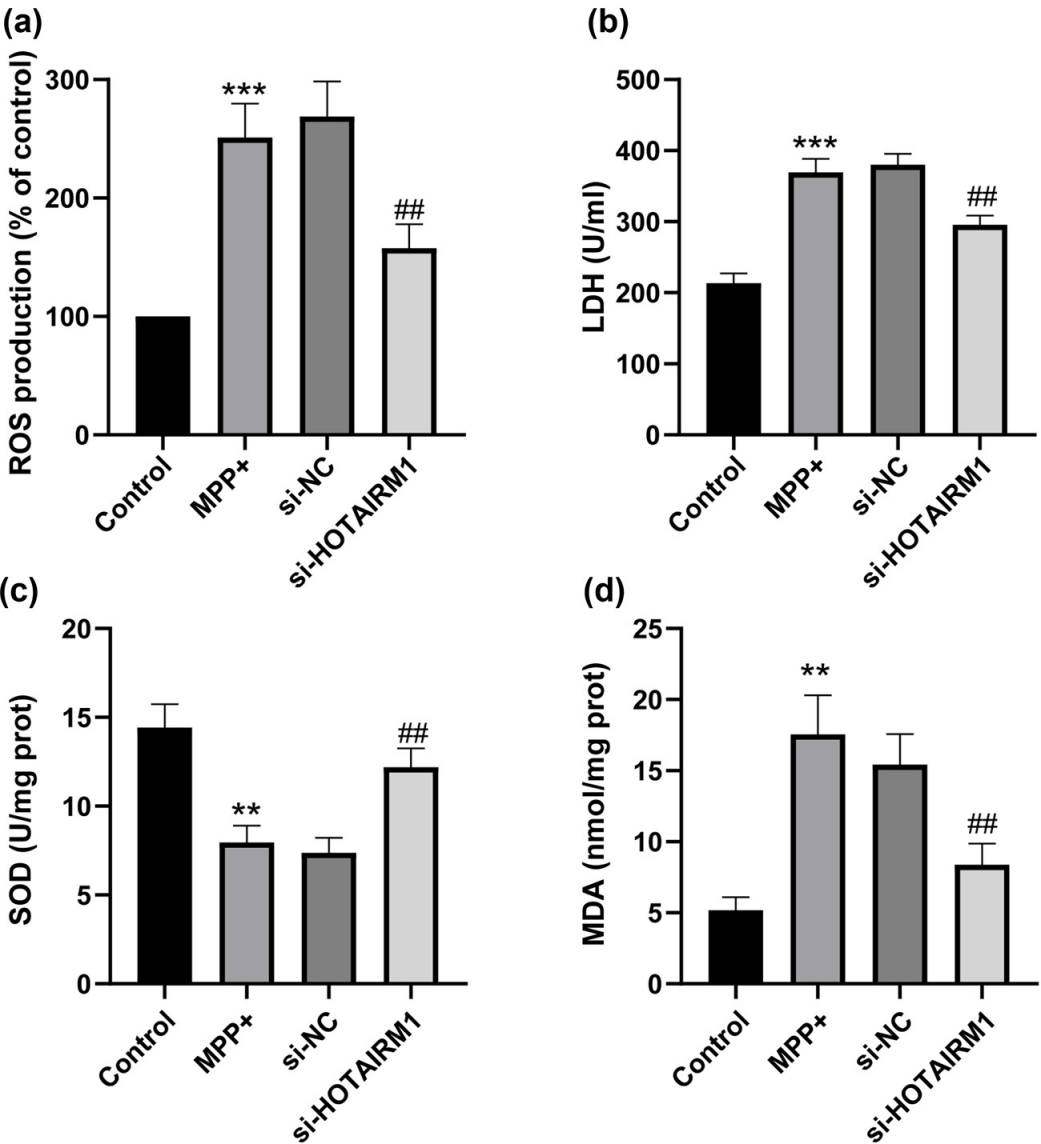
HOTAIRM1 knockdown alleviates MPP^+^-induced SH-SY5Y cell oxidative stress injury. SH-SY5Y cells were transfected with si-NC and si-HOTAIRM1MPP^+^ for 24 h before cells were challenged by 1.0 mM MPP^+^ for 24 h. (a) ROS level in each group. (b) LDH activity in each group. (c) SOD level in each group. (d) MDA level in each group. Compared with the control group, **p < 0.01, ***p < 0.001; compared with the si-NC group, ^#^ *p* < 0.05, ^##^ *p* < 0.01.

To further elucidate the potential mechanism of HOTAIRM1, the effect of HOTAIRM1 in SH-SY5Y cell oxidative stress was evaluated (all *p* < 0.01, Figure 4(a) and (d)). ROS, LDH, and MDA levels were elevated in SH-SY5Y cells treated with MPP^+^, and SOD level was downregulated (all *p* < 0.01), while HOTAIRM1 knockdown led to reversed results (all *p* < 0.01), suggesting that HOTAIRM1 downregulation rescued MPP^+^-induced SH-SY5Y cell oxidative stress injury.

### 3.5 HOTAIRM1 knockdown activates the Nrf2/HO-1 pathway in MPP^+^-induced SH-SY5Y cells

**Figure 5:**
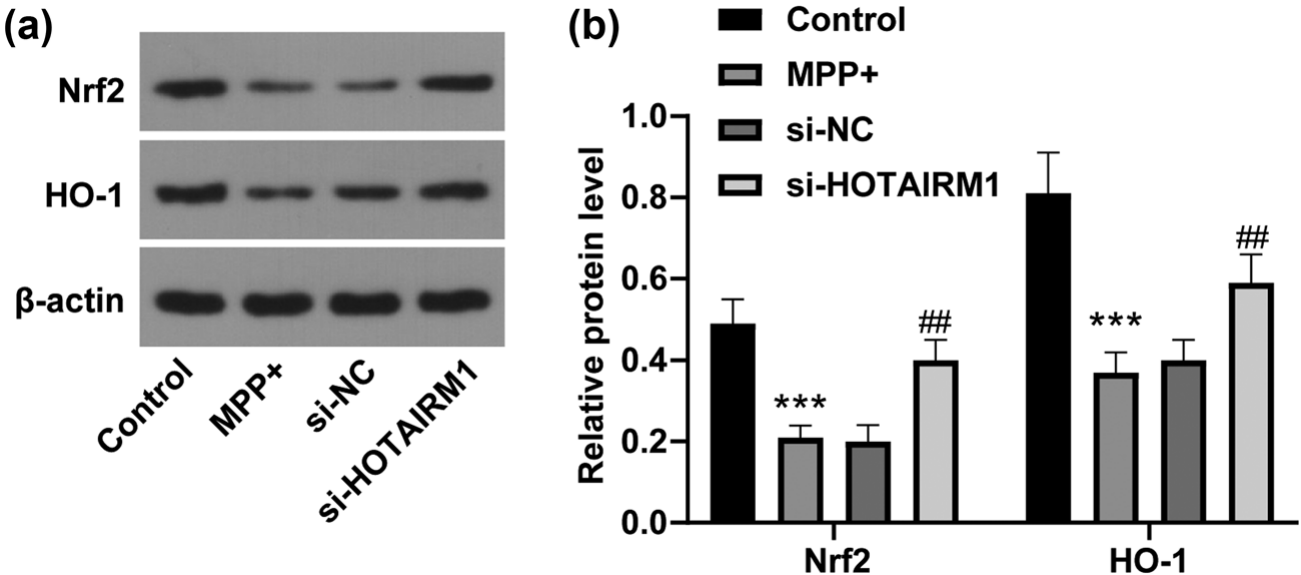
HOTAIRM1 knockdown could activate the Nrf2/HO-1 pathway in MPP^+^-induced SH-SY5Y cells. SH-SY5Y cells were transfected with si-NC and si-HOTAIRM1MPP^+^ for 24 h before cells were challenged by 1.0 mM MPP^+^ for 24 h. (a) Western blot analysis was conducted to assess the protein levels of Nrf2 and HO-1. (b) Comparison of the gray values of Nrf2 and HO-1. Compared with the control group, ***p < 0.001; compared with the si-NC group, ^##^ *p* < 0.01.

The Nrf2/HO-1 pathway mitigates oxidative stress injury [22,23]. To figure out whether HOTAIRM1 knockdown could activate the Nrf2/HO-1 pathway in SH-SY5Y cells, levels of oxidative stress proteins (Nrf2 and HO-1) were detected via western blot analysis (Figure 5(a) and (b)), which found that compared with that in the control group, the MPP^+^ group showed decreased protein levels of Nrf2 and HO-1 (all *p* < 0.001), and the si-HOTAIRM1 group had increased protein levels of Nrf2 and HO-1 (all *p* < 0.01). In conclusion, HOTAIRM1 knockdown could activate the Nrf2/HO-1 pathway in MPP^+^-induced SH-SY5Y cells.

### 3.6 Nrf2 inhibition counteracts the role of HOTAIRM1 knockdown in MPP^+^-induced SH-SY5Y cell oxidative stress

**Figure 6:**
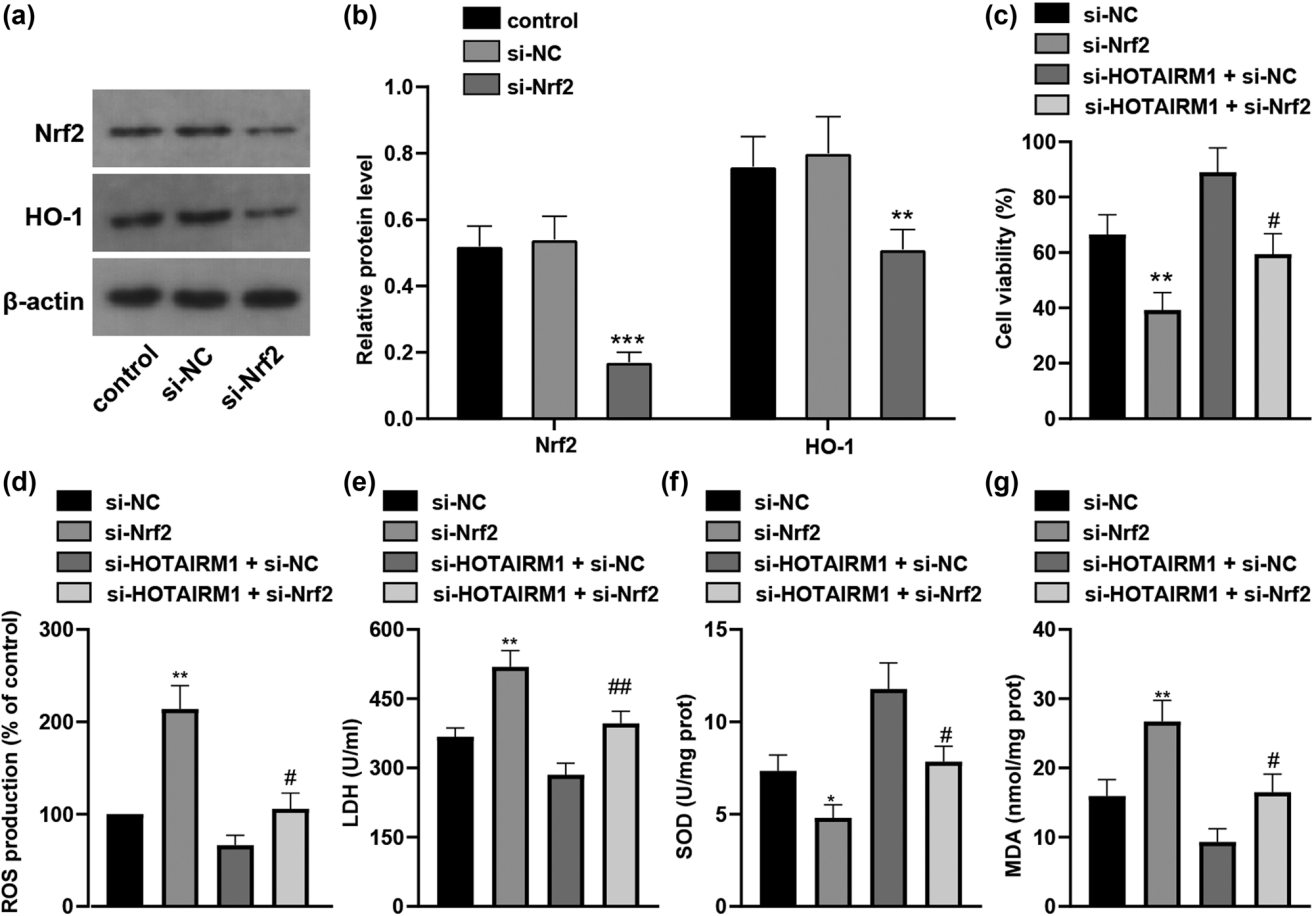
Nrf2 inhibition counteracts the role of HOTAIRM1 knockdown in MPP^+^-induced SH-SY5Y cell oxidative stress. SH-SY5Y cells were transfected with si-NC and si-HOTAIRM1MPP^+^ for 24 h before cells were challenged by 1.0 mM MPP^+^ for 24 h. (a) Western blot analysis was conducted to assess the protein levels of Nrf2 and HO-1. (b) Comparison of the gray values of Nrf2 and HO-1. Compared with the si-NC group, ***p < 0.001, **p < 0.01. (c) Cell viability was measured by CCK-8 assay. (d) ROS level in each group. (e) LDH activity in each group. (f) SOD level in each group. (g) MDA level in each group. Compared with the si-NC group, **p < 0.01; compared with the si-HOTAIRM1 + si-NC group, ^#^ *p* < 0.05.

To elucidate the role of the Nrf2/HO-1 pathway in the *in vitro* PD cell model, si-Nrf2 was applied to silence Nrf2, so as to explore the role of HOTAIRM1 knockdown in the Nrf2/HO-1 pathway in MPP^+^-induced SH-SY5Y cell oxidative stress (all *p* < 0.01, Figure 6(a) and (b)). The results from CCK-8 assay and oxidative stress index assessment unveiled that si-Nrf2 neutralized the suppressive role of HOTAIRM1 knockdown in cell viability (*p* < 0.05, Figure 6(c)) and oxidative stress index levels (all *p* < 0.05, Figure 6(d)–(g)). All in all, HOTAIRM1 modulated oxidative stress in the *in vitro* PD cell model via the Nrf2/HO-1 pathway.

## 4 Discussion

As a prevalent age-dependent disease, PD could be triggered by both environmental and genetic reasons and it imposes movement impairment and neurological function deficit on patients [24]. lncRNAs are the significant players in PD pathogenesis by affecting various pathological mechanisms, including apoptosis, neuroprotection, cellular functions, and oxidative stress injury [25]. Robustly expression of HOTAIRM1 was necessarily connected to PD expansion [26], illustrating that HOTAIRM1 might be a detrimental cytokine in PD progression. Our experiment was designed to probe potential treatment for PD with the involvement of HOTAIRM1. Collectively, we found that HOTAIRM1 knockdown could suppress oxidative stress damage in the *in vitro* PD model via the Nrf2/HO-1 pathway.

MPP^+^ treatment accelerated oxidative stress damage, cell death, cytotoxicity, and neuroinflammation in PD [27]. Besides, SH-SY5Y cells have become the well-accepted choice for PD cell model establishment for their DAergic neuronal properties and sensitivity to neuroprotection and cytotoxicity [28]. In our experiment, SH-SY5Y cells were treated with MPP^+^ to establish a PD cell model, from which we noticed that HOTAIRM1 expression was upregulated. The variance of HOTAIRM1 expression in different neoplasms could control cancer cellular biological activities, making HOTAIRM1 a popular molecule in tumor therapy [12]. Besides, HOTAIRM1 was highly expressed in allergic rhinitis, thereby exaggerating the adverse reactions triggered by allergen [29]. Despite the accumulating research on the effects of HOTAIRM1 in human diseases, the specific mechanism of HOTAIRM1 in PD is to be explored. To figure out the effect of HOTAIRM1 on MPP^+^-induced SH-SY5Y cell viability, si-HOTAIRM1 was transfected into SH-SY5Y cells, after which cell viability was enhanced and cytotoxicity was reduced. HOTAIRM1 interferes cytotoxicity in acute myeloid leukemia to alter tumor progression [30]. Remarkably, HOTAIRM1 expression was elevated in the *in vitro* PD model [15]. To sum up, HOTAIRM1 could be a detrimental cytokine in the SH-SY5Y cell model.

Apoptosis is a crucial mechanism in PD mitigation as it results in neuron impairment, exaggerates mitochondrial function loss, and elicits inflammatory damages [31]. To elaborate the role of HOTAIRM1 in MPP^+^-induced SH-SY5Y cell apoptosis was researched. TUNEL assay was conducted, and it found that HOTAIRM1 knockdown led to suppressed apoptosis with the involvement of downregulated Bax level and upregulated Bcl-2 level. Bax encouraged neural apoptosis and cytotoxicity aggravated by MPP^+^ treatment [32]. Furthermore, Bcl-2 could modulate apoptotic reactions and maintain mitochondrial balance, thereby reducing Parkinsonian syndrome [33]. It was recently reported that HOTAIRM1 elicited apoptosis as presented by increased Bax expression and decreased Bcl-2 expression in carcinomas [34]. Therefore, HOTAIRM1 knockdown inhibited MPP^+^-induced SH-SY5Y cell apoptosis. On the other hand, oxidative stress spoils neuronal environment and accelerates dopamine exhaustion in PD patients [35]. To further elucidate the potential mechanism of HOTAIRM1 in oxidative stress, it was silenced in SH-SY5Y cells, and then, we observed that ROS, LDH, and MDA levels were downregulated, and SOD level was elevated. Significantly, in experimental models with PD, increased level of SOD and suppressed levels of ROS, LDH, and MDA were associated with reduced oxidative stress damages, inhibited inflammatory injuries, and discouraged neurodegeneration [36]. Silencing of HOTAIRM1 could also sabotage levels of ROS, LDH, and MDA while promoted SOD expression [16]. Additionally, HOTAIRM1 downregulation rescued MPP^+^-induced SH-SY5Y cell oxidative stress injury.

The Nrf2/HO-1 pathway protected neurons from degenerative reactions and promoted mitochondrial homeostasis in PD [37]. To figure out whether HOTAIRM1 knockdown could activate the Nrf2/HO-1 pathway in SH-SY5Y cells, HOTAIRM1 was silenced, after which it was unraveled that protein levels of Nrf2 and HO-1 were increased. HOTAIR and Nrf2 were often negatively connected, and lowly expressed HOTAIR and overexpressed Nrf2 evidently attenuated cytotoxicity [38]. Collectively, HOTAIRM1 knockdown could activate the Nrf2/HO-1 pathway in MPP^+^-induced SH-SY5Y cells. Mechanically, Nrf2 and HO-1 contributed to strengthened cellular viability and limited apoptosis in anoxic conditions [39]. Besides, Nrf2 is deemed as a powerful molecule in defending cells from cytotoxicity and oxidative stress damages [40]. To elucidate the role of the Nrf2/HO-1 pathway in the *in vitro* PD model, Nrf2 was silenced, and our results revealed that Bax expression was encouraged, Bcl-2 expression was discouraged, level of SOD was improved, and levels of ROS, LDH, and MDA were inhibited. When Nrf2 and HO-1 were overexpressed, ROS, LDH, and MDA levels were downregulated to restrict cell damage, dysfunction, and death [41]. The Nrf2/HO-1 pathway could reverse apoptotic reactions, inflammation, and oxidative stress damages as evidenced by elevated SOD and Bcl-2 levels and reduced Bax expression in acute lung injury [42]. In conclusion, HOTAIRM1 modulated oxidative stress in the *in vitro* PD model via the Nrf2/HO-1 pathway.

## 5 Conclusion

All in all, our study clarified that HOTAIRM1 knockdown alleviated MPP^+^-induced SH-SY5Y cell oxidative stress injury via the Nrf2/HO-1 pathway, laying the theoretical foundation for the study of PD treatment and pathogenesis. However, the role of HOTAIRM1 knockdown was only researched in the cell model, which may be different from the animal model or human body. What is more, the specific molecular mechanism of HOTAIRM1 oxidative stress still needs to be further investigated to provide a more theoretical basis for the translational therapeutic application of PD.
